# Prediction and detection of freezing of gait in Parkinson’s disease from plantar pressure data using long short-term memory neural-networks

**DOI:** 10.1186/s12984-021-00958-5

**Published:** 2021-11-27

**Authors:** Gaurav Shalin, Scott Pardoel, Edward D. Lemaire, Julie Nantel, Jonathan Kofman

**Affiliations:** 1 Department of Systems Design Engineering, University of Waterloo, Waterloo, ON Canada; 2 Faculty of Medicine, University of Ottawa and Ottawa Hospital Research Institute, Ottawa, ON Canada; 3 School of Human Kinetics, University of Ottawa, Ottawa, ON Canada

**Keywords:** Freezing of gait, Parkinson’s disease, Plantar pressure, Long short-term memory, Deep learning, Detection, Prediction

## Abstract

**Background:**

Freezing of gait (FOG) is a walking disturbance in advanced stage Parkinson’s disease (PD) that has been associated with increased fall risk and decreased quality of life. Freezing episodes can be mitigated or prevented with external intervention such as visual or auditory cues, activated by FOG prediction and detection systems. While most research on FOG detection and prediction has been based on inertial measurement unit (IMU) and accelerometer data, plantar-pressure data may capture subtle weight shifts unique to FOG episodes. Different machine learning algorithms have been used for FOG detection and prediction; however, long short-term memory (LSTM) deep learning methods hold an advantage when dealing with time-series data, such as sensor data. This research aimed to determine if LSTM can be used to detect and predict FOG from plantar pressure data alone, specifically for use in a real-time wearable system.

**Methods:**

Plantar pressure data were collected from pressure-sensing insole sensors worn by 11 participants with PD as they walked a predefined freeze-provoking path. FOG instances were labelled, 16 features were extracted, and the dataset was balanced and normalized (z-score). The resulting datasets were classified using long short-term memory neural-network models. Separate models were trained for detection and prediction. For prediction models, data before FOG were included in the target class. Leave-one-freezer-out cross validation was used for model evaluation. In addition, the models were tested on all non-freezer data to determine model specificity.

**Results:**

The best FOG detection model had 82.1% (SD 6.2%) mean sensitivity and 89.5% (SD 3.6%) mean specificity for one-freezer-held-out cross validation. Specificity improved to 93.3% (SD 4.0%) when ignoring inactive state data (standing) and analyzing the model only on active states (turning and walking). The model correctly detected 95% of freeze episodes. The best FOG prediction method achieved 72.5% (SD 13.6%) mean sensitivity and 81.2% (SD 6.8%) mean specificity for one-freezer-held-out cross validation.

**Conclusions:**

Based on FOG data collected in a laboratory, the results suggest that plantar pressure data can be used for FOG detection and prediction. However, further research is required to improve FOG prediction performance, including training with a larger sample of people who experience FOG.

## Background

Freezing of gait (FOG) is a gait disorder seen in some individuals in the early stage of Parkinson’s disease (PD) and in up to 60% of people in the more advanced stages of the disease [1–5]. FOG is characterized by the inability to step effectively and move forward despite the intention to do so. FOG has been shown to cause falls [5], even when the FOG episode is brief (less than 10 s) [6]. Even in non-fallers, repeated freeze episodes can negatively affect overall mobility, level of activity, and thus their independence and quality of life [7–10]. Therefore, reducing FOG occurrence can greatly improve independence and quality of life.

Cueing can alleviate FOG by providing an external stimulus, such as light or sound, that facilitates gait initiation and continuation [11]. However, continuous cueing may be distracting when a person is not walking, and can lead to cue dependency [12] and induce fatigue [13]. Furthermore, cueing with a pre-set rhythm that is not matched to the person’s specific gait at each instant may induce FOG [14]. It is therefore preferred to use an intelligent cueing approach [15] that could activate a cue upon freeze detection (in order to end the freeze and reduce freeze duration), or ideally, predict a freeze before onset and activate pre-emptive cueing (to prevent the freeze from occurring). Development of freeze detection and prediction methods are therefore important for an intelligent cueing system designed to reduce freeze duration and occurrence.

Most FOG detection research has used accelerometer or inertial measurement unit (IMU) sensors [16–22] at the ankle, knee, and waist. Plantar pressure has been used in rehabilitation strategies [23–26], fall-risk prediction [27] and classification [28] in older adults, and classifying gait as PD or healthy control [29]. Only recently, plantar pressure data has been used together with accelerometer data for FOG detection [30], and in our recent research for early FOG detection and prediction (together with IMU data [31, 32] and alone [32, 33]). Plantar pressure data was useful in detecting FOG and the transition from normal walking into a freeze. Since an integrated shoe-based plantar-pressure system may be less obtrusive and easier to wear than accelerometer and IMU sensors placed on various parts of the body, a FOG detection and prediction system based on plantar-pressure-sensing insoles could permit greater user compliance than a system that uses accelerometer or IMU sensors on multiple body parts. There is therefore a need to determine the effectiveness of FOG detection and prediction methods based on plantar-pressure sensors alone.

Many machine-learning algorithms have been used for classifying FOG [16, 19, 20, 34]. Random forests (in both participant dependant [16] and independent models [20]) and Support Vector Machines (SVM) [19, 34] have performed well in FOG classification. However, Recurrent Neural Networks (RNN) have the potential to achieve better performance due to the way time-series data are handled. RNN are artificial neural networks that can model sequence data (including time-series data from wearable sensors), taking a sequence as input, and outputting a sequence. Long short-term memory (LSTM) units were introduced to solve the vanishing gradients problem in RNN, and thus allow RNN to learn from longer sequences [35]. When trained with acceleration data, a LSTM network reported 83.38% (SD 10%) FOG detection accuracy [17]. To improve the existing FOG detection models, efforts have been made to detect FOG earlier (i.e., predict the FOG episode before it occurs) using FOG indicators found in gait parameters preceding a freeze episode [36]. These gait parameters showed that gait can deteriorate as the person progresses into a freeze. This period of gait deterioration preceding a freeze is often referred to as Pre-FOG and has been used for FOG prediction using various methods [37–39]. Recently, LSTM networks have also been used for FOG prediction. In [40], a 2-layer LSTM network was used for a 2-class model with Pre-FOG and FOG classes together in the target class. The LSTM network achieved 87.54% accuracy for 1 s Pre-FOG duration, 85.54% accuracy for 3 s Pre-FOG duration, and 79.47% accuracy for 5 s Pre-FOG duration, all using acceleration signals [40]. However, with an imbalanced dataset, the study gave a biased analysis since only accuracy was reported [40]. In the case of an imbalanced dataset, metrics such as sensitivity and specificity should be used instead of accuracy since many true negatives can increase accuracy regardless of the model’s ability to produce true positives.

The existing research has established a foundation for FOG detection and prediction; however, several limitations need to be addressed. For instance, most research has focused on participant dependent models [16, 17, 38, 40, 41]. Since these models were not validated on unseen participant data, applicability to a new person with PD is limited. Furthermore, participant dependent models give biased results due to a correlation between training and validation samples. This limitation has been acknowledged, but not completely mitigated [16, 41]. Some FOG detection [18, 21] and FOG prediction [42] models have been validated on a set of held-out participants; however, model performance standard deviation across different held out participants was not reported. Another limitation, specific to deep learning models, is a large batch size (e.g., 128 [43] or 1000 [40]) that can lead to poor generalization. A batch size of 1 has been recommended to minimize generalization error [44], the drawback being that with a batch size of 1, the network will take more time to converge to the global optimum.

A FOG detection model should require minimal preprocessing (including signal filtering) without any manual steps, to reduce freeze detection latency and permit the model to be run in real-time on a wearable microcontroller. The model should also require minimal computer memory. However, most research used noise filtering [20, 39, 42], manual steps [20], preprocessing such as combining windows in the spectral domain [18, 21], or computationally intensive feature extraction [19, 34] before FOG detection or prediction, and only one study has focused on finding and reducing freeze detection latency [16].

Plantar-pressure-sensing insoles could be used to make integrated shoe-based FOG detection and prediction systems that could permit greater user compliance than accelerometer or IMU based systems that require multiple sensors on various body locations. For this reason, this research investigated the effectiveness of FOG detection and prediction methods based on plantar pressure sensors. The ability to utilize time series data makes RNN, and especially LSTM networks, well suited to FOG detection and prediction. The aim of this research was to demonstrate the effectiveness of LSTM models for FOG detection and prediction while maintaining generalizability by using leave one participant out (LOPO) cross validation and model training with a batch size of 1.

## Methods

### Participants and inclusion criteria

A convenience sample of 11 male participants was recruited from the Ottawa-Outaouais community, with mean age 72.7 years (SD 5.5), height 1.77 m (SD 0.04), weight 79.6 kg (SD 10.5), and 10.5 years (SD 4.8) since PD diagnosis. Eligibility criteria were: PD with FOG at least once a week, able to walk 25 m unassisted (without a cane or walking aid) and no lower limb injury or other comorbidity that impaired ability to walk. In addition, participants must not have undergone deep brain stimulation therapy. Participants visited the lab for a single data collection session while on their normal antiparkinsonian medication dosage and schedule. Data collection was typically scheduled in the hours prior to the participant’s next dose so that the medication would be wearing off during testing and FOG would be more likely to occur. After providing written informed consent, all participants took part in a clinical assessment which included the New Freezing of Gait questionnaire, a self-reported fall history questionnaire, and the Motor Examination section from the Unified Parkinson’s Disease Rating Scale (UPDRS III). Participants also completed an information form that included disease history and medication schedule.

### Plantar pressure measurement

**Fig. 1:**
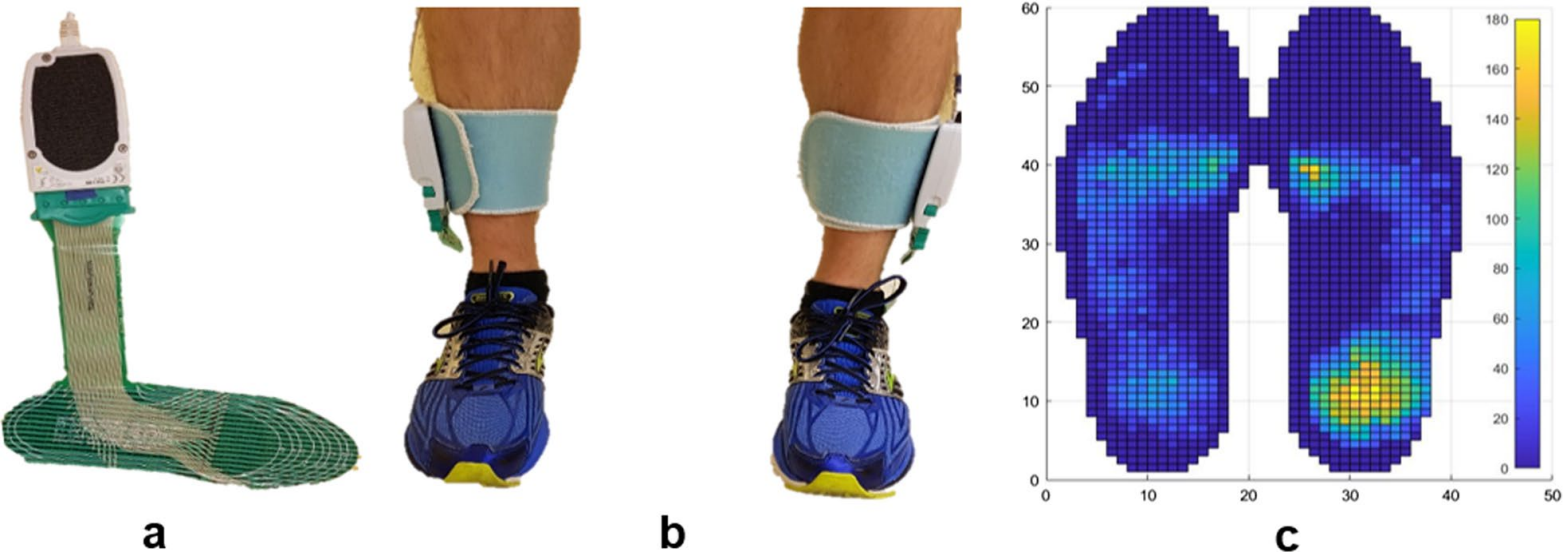
F-Scan system: **a** single plantar pressure insole sensor, **b** sensors worn in shoes, and **c** plantar pressure sample frame (kPa); dark blue indicates zero pressure

Plantar pressure data were recorded during multiple walking trials at 100 Hz using FScan pressure sensing insoles (Tekscan, Boston, MA; Fig. 1). FScan insoles are thin (< 1 mm) plastic film sheets with 3.9 pressure sensing cells per cm^2^ (25 cells per in^2^). The insoles were equilibrated before participant arrival by applying uniform pressure to the entire sensor and adjusting the sensor constants to produce a uniform output [45]. Equilibration was performed at 138 kPa, 276 kPa, and 414 kPa. A sample plantar-pressure data frame from both feet is shown in Fig. 1c.

### Test protocol

**Fig. 2:**
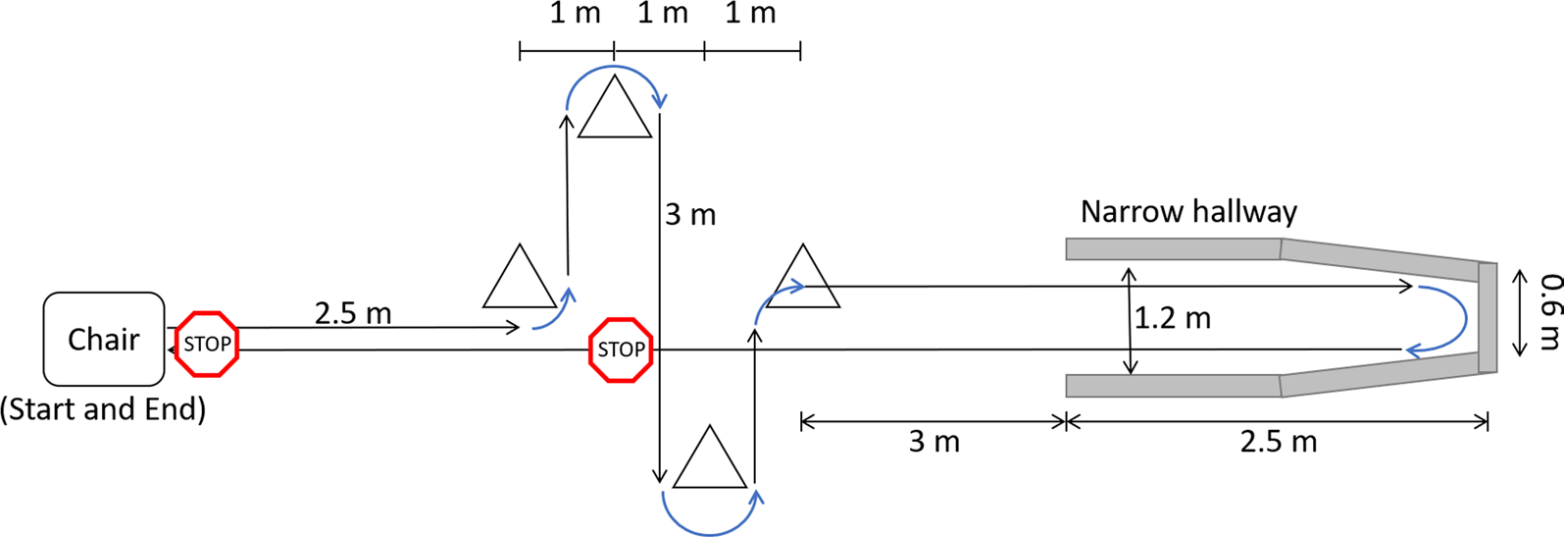
Walking Path

Prior to data collection, participants were weighed, and the plantar pressure sensors were trimmed and fitted into their shoes. A step calibration was performed (i.e., standing on one leg and then quickly transferring weight to the other foot, to calibrate sensors to body weight). Participants were asked to walk a pre-defined path (approximately 25 m) that involved navigating multiple cones (requiring two 90° and two 180° turns); walking as far as possible into a narrow, dead-end hallway (2.1 m tall, 1.2 m wide, 2.5 m long); turning 180°; and walking back to the starting position (Fig. 2). Participants were asked to stop once in a place of their choice in the area delimited by the cones (voluntarily stop) while walking back to the starting position. Participants were also required to stop in front of the chair at the end of each test trial (prescribed stop).

**Fig. 3:**
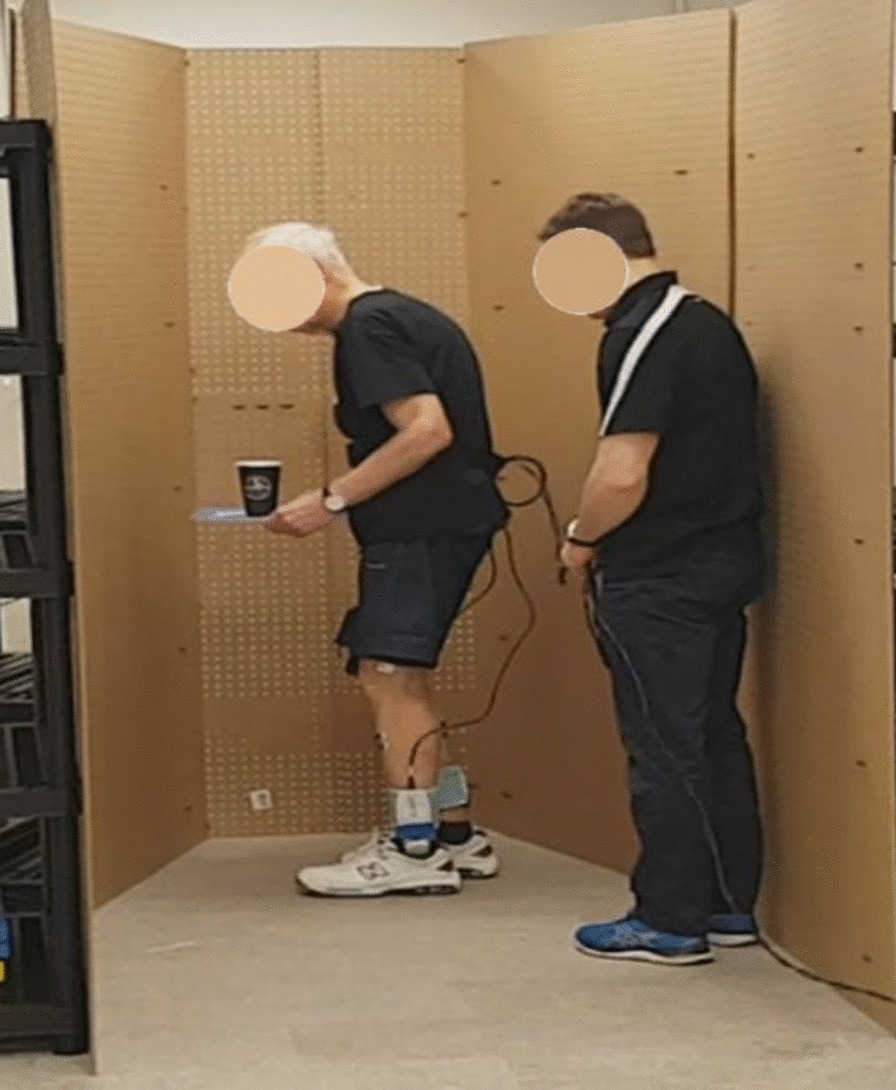
PD participant turning in a narrow hallway while holding a tray with a cup. Assistant follows for safety

Walking trials were recorded using a smartphone camera so that freezing instances could be labeled following data collection. For each walking trial, participants stood up from a sitting position and performed a single foot stomp before starting to walk. The stomp was later used to synchronize plantar pressure data and video. Participants completed up to 30 trials. The first five were baseline trials, where participants completed the walking path without any additional tasks, after which, additional tasks were added if the participant did not freeze (Fig. 3). These tasks were verbal (continuously speaking as many words as possible beginning with a specific letter) and motor (holding a plastic tray with both hands, with objects on the tray), performed individually or simultaneously. Different difficulty levels were used when performing the motor task, for example, starting with three small wooden blocks on the tray and adding additional blocks as needed, to increase difficulty. Alternatively, the blocks were replaced with an empty paper coffee cup or sealed water bottle, or the participant was asked to carry the tray with only one hand.

### Data preprocessing

Plantar pressure data were labeled as FOG and non-freeze (Non-FOG), using a custom-made MATLAB R2019b application (MathWorks, MA, USA). During data collection, authors SP and JN identified FOG occurrences. In post processing, SP identified the onset and termination of FOG episodes from the video recording to a resolution of 30 Hz. In case of uncertainty, the second rater was consulted. The beginning of a freeze was defined as “the instant the stepping foot fails to leave the ground despite the clear intention to step”. The end of the freeze was defined as “the instant the stepping foot begins or resumes an effective step”. For example, a step was considered effective at the instant the heel lifted from the ground, provided that it was followed by a smooth toe off with the entire foot lifting from the ground and advancing into the next step without loss of balance. The labels were applied to the plantar pressure data using linear interpolation to the closest timestamp, such that each individual datapoint had a label. Subsequently, for the FOG prediction analysis, some data preceding each freeze were labeled as Pre-FOG. At each instant in time (i.e., each datapoint), the plantar pressure data (Fig. 1c) consisted of two 60 × 21 matrices of pressure values, one for each foot. Each walking trial was kept as a unique time series wherein each datapoint had a distinct timestamp and label. This preserved the time series information that would be lost if data from any two trials were concatenated or mixed. Plantar pressure data from the left and right foot were kept separate.

A set of 16 features were extracted from the plantar pressure data:

- Centre of pressure coordinates (mm): Centre of pressure (COP) coordinates were converted to distance from the origin by multiplying the cell position by the distance between two cells (5.08 mm). COP coordinates in *x* (medial/lateral) and *y* (anterior/posterior) directions for both feet were calculated at each timestamp.
- Centre of pressure velocity (cm/s): COP velocity was calculated by dividing the COP coordinates difference between two consecutive samples by the time difference between the samples (0.01 s). COP velocity was calculated for both *x* and *y* axes for both feet.
- Centre of pressure acceleration (cm/s^2^): COP acceleration was calculated by dividing the COP velocity difference between two consecutive samples by the time difference between samples. COP acceleration was calculated for both *x* and *y* axes for both feet at each timestamp.
- Total ground reaction force (kPa): Total Ground Reaction Force (GRF) was obtained by adding pressure from all pressure cells of the plantar pressure sensor. Total GRF was determined for both feet at each timestamp.
- Fraction of total ground reaction force (unitless): Fraction of total GRF is the ratio of GRF from one foot divided by the total GRF for both feet. Fraction of total GRF was calculated for both feet at each timestamp.

COP coordinates, COP velocity, and COP acceleration were calculated for both *x* and *y* axes for both feet. Total GRF and fraction of total GRF were calculated for both feet. The features were calculated at each timestamp and were used for FOG detection and prediction.

Each time a model was trained, each participant was assigned to either the training or the testing set (i.e., for any given participant, the participant data were either entirely in the training set, or entirely in the validation set).

### Data balancing for detection models

Since FOG events occur much less frequently than regular walking steps, the dataset was imbalanced, with most walking trials consisting mostly of Non-FOG data (walking data without a FOG episode). A custom data balancing approach was designed to account for this imbalance, creating one training instance for each FOG episode in the training dataset. A training instance was defined as a single FOG episode with some Non-FOG data before and after the freeze. If possible, the ratio of FOG to Non-FOG data in each training instance was 1:1. The amount of Non-FOG data taken before and after the FOG episode each corresponded to half the duration of the FOG episode. If there was not enough Non-FOG data on one side of a FOG episode (e.g., if the FOG episode occurred at the beginning or end of a trial), then additional Non-FOG data was taken on the other side of the freeze such that the total amount of Non-FOG data was equal to the amount of FOG data in the training instance. However, in a few cases, there were not enough Non-FOG data points before and after a FOG event (this happened when multiple FOG episodes occurred close to each other). In such cases, the FOG episode along with the available Non-FOG data between freezes was extracted. Therefore, most training instances had equal numbers of FOG and Non-FOG datapoints. A few slightly imbalanced training instances had more FOG datapoints than Non-FOG (when multiple FOG occurred in rapid succession). Data between FOG episodes could be included after the FOG in one classification instance or before the FOG in a different classification instance, and if necessary, the same Non-FOG data could be used for both cases. For the test set, no balancing was performed since the model is intended to be used in real-time situations where the input data would be imbalanced.

### LSTM model

For FOG detection, LSTM networks were setup using a multiple-input (multiple datapoint) multiple-output (multiple datapoint) architecture in which all datapoints were used as model inputs and each datapoint in the test instances was classified by the model. Each LSTM layer returned the full sequence to the model’s next layer. This allowed the model to classify each timestamp as belonging to the FOG or Non-FOG class. LSTM layers used a hyperbolic tangent (*tanh*) activation function, followed by a time-distributed fully connected layer (i.e., output at each time step passes through the fully connected layer) with 2 units and Softmax activation. Models were trained with the Adam optimizer, using 0.9 decay rate for the first and 0.999 decay rate for the second moment estimates, and a cross entropy loss function.

Most deep learning frameworks (e.g., TensorFlow) require that all sequences in the same batch have the same length for vectorization. Vectorization treats network weights and inputs as vectors, allowing vector multiplication rather than repetitive element-wise multiplications. Sequences of different lengths can be handled by using a batch size of 1. For evaluation, the total number of correct and incorrect classifications (one classification per datapoint) in the validation set (i.e., a held-out participant’s data) were used to calculate the model’s specificity and sensitivity. The model precision and F1 score (harmonic mean of sensitivity and precision) were also calculated.

### Hyperparameter tuning

Several network architectures and learning rate combinations were tried while using the Adam optimizer, cross entropy loss function, and a batch size of 1 (Table 1). All models had a time-distributed fully connected layer with 2 neurons and a Softmax activation after the LSTM layers. For this stage of model development, the training and test sets were fixed, Participant 2 was used as the held-out test set and all other freezers were used for training.

**Table 1:** LSTM Network configurations

Hyperparameter	Values tested
Number of LSTM layers	1, 2, 3, 4, 5
Number of units in each LSTM layer	16, 32, 64
Constant learning rate	0.1, 0.01, 0.001, 0.0001
Learning rate decay with a decay rate (decay rate, initial learning rate)	(0.5, 0.005), (0.75, 0.001)
Learning rate decreases in discrete steps (initial learning rate)	Decreases to half every 5 epochs (0.01)

**Fig. 4:**
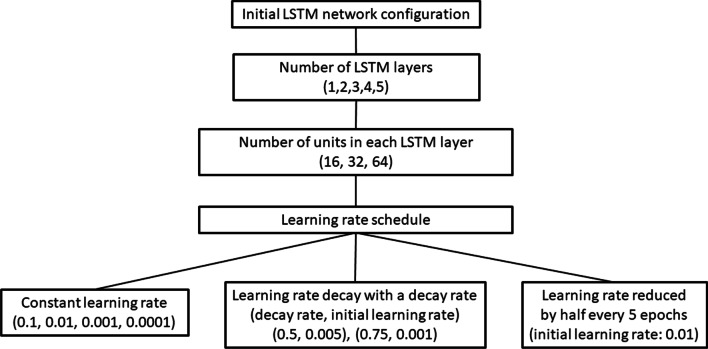
Hyperparameter tuning of the LSTM network architecture

An initial 2-layer LSTM network was trained with 16, 32, or 64 units in both layers for 30 epochs with a 0.01 constant learning rate. The network worked best using 16 units in each layer. The 2-layer LSTM network’s performance did not improve beyond 30 epochs. Thus, in all subsequent experiments, the network was trained only until 30 epochs. Then, networks with 16 units in each layer, were trained using 1, 2, 3, 4, or 5 LSTM layers. Networks with 1, 4, or 5 LSTM layers performed poorly. Networks with 2 or 3 LSTM layers performed best. It was thought that the 3 layer-LSTM network may outperform the 2-layer network if model complexity were further increased by using more units per layer; therefore, the number of units was varied for the 3-layer network. For the 3 LSTM layer model, 32 units in each layer performed better than 16 units in each layer. Only networks with 2 LSTM layers (each with 16 units) and 3 LSTM layers (each with 32 units) were used for subsequent experiments. A schematic representation of the hyperparameter tuning of the LSTM model is shown in Fig. 4.

Different learning rates were explored for the 2 and 3-layer LSTM network; 0.1, 0.01, 0.001, and 0.0001 constant learning rates. Learning rate decay with 0.5 decay rate (0.005 initial learning rate) and 0.75 decay rate (0.001 initial learning rate) were used (Fig. 4). A learning rate schedule where learning rate was reduced to half every 5 epochs, after starting from a 0.01 initial learning rate, outperformed all other learning rate schedules. The best performing models were used for FOG detection (Table 2).

**Table 2:** Best performing LSTM network configurations for FOG detection

Network or training parameter	Values / Options
LSTM layers (units in each LSTM layer)	2 layers (16 units) and 3 layers (32 units)
Initial learning rate	0.01
Learning rate decay	Decreases to half, every 5 epochs
Optimizer	Adam optimizer
Loss function	Cross entropy loss function
Batch size	1
Training epochs	30

### Cross validation

The best performing models (Table 2) were trained and evaluated with cross validation after z-score normalization as follows:

- One-freezer-held-out cross validation: The model was trained on data from all but one participant who froze during trials and was validated on the held out participant. This was repeated for each freezer, such that each freezer was in the validation set once.
- All-non-freezers-held-out validation: The model was trained on data from all participants who froze and was validated on all participants who did not freeze during the trials. This facilitated false positive assessment in situations where participants did not freeze.

The mean and standard deviation of each feature were calculated on the entire training set and then used for normalizing each corresponding feature in both the training and validation sets. Each feature was normalized using z-score normalization by subtracting the feature value by the feature’s mean and dividing by the feature’s standard deviation. Each time a new participant was held out, the z-score normalization was re-done using the mean and SD of the current training set. Z-score normalization is useful for removing outliers and bringing all features to a similar scale.

### FOG detection latency

FOG detection latency is the time difference between freeze onset and the detection of the freeze by the model. A freeze episode was detected correctly if the model classified a freeze during the true freeze period (period between the freeze onset and end of the freeze episode). If multiple freeze classifications existed within the true freeze period, the earliest classification was used to calculate freeze detection latency.

### FOG prediction

For FOG prediction, the LSTM model architecture that was best for FOG detection, and the same validation approaches (one-freezer-held-out, all-non-freezers-held-out) were utilized. The primary difference between the detection and prediction models was the dataset labeling. FOG prediction is usually a three-class classification problem, with the classes being FOG, Pre-FOG (data immediately prior to a freeze), and Non-FOG. FOG prediction has also been done as a binary classification [40], where data just before a freeze episode (Pre-FOG) and the freeze episodes were in the target class (i.e., class that the model aims to detect) and the remaining Non-FOG data were in the non-target class. The same binary classification setup was used for FOG prediction in this research. The data length before a freeze (Pre-FOG duration) depended on the corresponding freeze episode’s length. A 2 s Pre-FOG duration was chosen for all freeze episodes that were 2 s or longer, and a Pre-FOG duration equal to the corresponding freeze episode length was chosen for all freeze episodes shorter than 2 s.

The same 16 features for FOG detection were used for FOG prediction. Data were split into training and validation sets by participant (i.e., all data from a participant were either in the training or validation set). To balance the training dataset, the procedure used for FOG detection was also used for FOG prediction, except that the target class for prediction had both Pre-FOG and FOG data. Therefore, when determining Non-FOG data to include in each training instance, the combined duration of the Pre-FOG and FOG data was matched by the duration of the Non-FOG data, when possible.

## Results

A total of 241 min of walking data were collected, during which seven participants froze (Table 3). This data included 362 freeze episodes, with most (221) freeze episodes corresponding to Participant 7. Following data balancing, much of the Non-FOG data were removed, which reduced the final size of the input dataset (Table 4).

**Table 3:** Freeze episode count and duration for each participant

Participant	Most affected side	Number of FOG episodes	Mean (SD) FOG duration (s)	Total FOG duration (s)
1	Right	49	0.69 (0.26)	34.05
2	Left	35	2.64 (1.61)	92.35
3	Left	14	1.06 (0.53)	14.88
4	Left	0	–	–
5	Right	0	–	–
6	Left	10	4.23 (3.80)	42.29
7	Right	221	1.52 (1.48)	336.20
8	Right	24	1.51 (1.05)	36.16
9	Left	9	0.75 (0.35)	6.74
10	Left	0	–	–
11	Right	0	–	–

**Table 4:** FOG detection model training data provided by each participant following data balancing

Participant	Number of training instances	Number of datapoints in Non-FOG class	Number of datapoints in FOG class
1	49	3432	3454
2	35	9253	9270
3	14	1492	1502
6	10	4235	4240
7	221	30,246	37,132
8	24	3381	3886
9	9	680	683

### FOG detection

The 2-layer LSTM model (detailed in Table 2) achieved 82.1% mean sensitivity (SD 6.2%) and 89.5% mean specificity (SD 3.6%) in one-freezer-held-out cross validation (Table 5). The model achieved 81.6% specificity in all-non-freezer-held-out validation (i.e., tested only on participants who did not freeze: Participants 4, 5, 10, 11). The 3-layer LSTM model achieved a slightly improved mean sensitivity (83.4%, SD 6.7%) but a slightly lower mean specificity (87.4%, SD 5.4%) than the 2-layer LSTM model in one-freezer-held-out cross validation (Table 6). In all-non-freezer-held-out validation, the 3-layer model improved the specificity to 87.7%. The 2-layer model achieved 25.3% (SD 18.6%) precision and 0.35 (SD 0.20) F1 score. Similarly, the 3-layer model achieved 23.2% (SD 18.8%) precision and 0.32 (SD 0.20) F1 score. The low values of precision and F1 may be attributed, in part, to the data imbalance in the test set.

**Table 5:** FOG Detection: One-freezer-held-out cross validation for the 2-layer LSTM model (16 units per LSTM layer)

Participant held out	FOG data	Non-FOG data	Sensitivity (%)	Specificity (%)	Precision (%)	F1 score
1	3454	82,943	83.0	92.5	31.6	0.46
2	9270	110,130	77.2	90.3	40.0	0.53
3	1502	142,157	72.5	92.8	9.6	0.17
6	4240	191,309	85.9	90.2	16.3	0.27
7	33,841	159,787	77.4	89.5	61.0	0.68
8	3640	101,415	86.2	81.2	14.1	0.24
9	683	141,373	92.2	89.8	4.2	0.08
Mean (SD)			82.1 ± 6.2	89.5 ± 3.6	25.3 ± 18.6	0.35 ± 0.20

**Table 6:** FOG Detection: One-freezer-held-out cross validation for the 3-layer LSTM model (32 units per LSTM layer)

Participant held out	FOG data	Non-FOG data	Sensitivity (%)	Specificity (%)	Precision (%)	F1 score
1	3454	82,943	83.6	86.3	20.2	0.33
2	9270	110,130	83.5	90.1	41.5	0.55
3	1502	142,157	71.7	92.0	8.6	0.15
6	4240	191,309	86.9	90.7	17.2	0.29
7	33,841	159,787	77.1	89.2	60.1	0.68
8	3640	101,415	87.6	74.7	11.1	0.20
9	683	141,373	93.6	88.6	3.8	0.07
Mean (SD)			83.4 ± 6.7	87.4 ± 5.4	23.2 ± 18.8	0.32 ± 0.20

Overall, the 2-layer and 3-layer models had similar performance, with a slight trade-off of lower specificity for higher sensitivity for the 3-layer model. However, the 2-layer model is much simpler, having fewer layers and fewer units per layer, and is therefore the better option for a wearable system. The simpler model is less prone to overfitting and has the additional advantage of lower computational cost.

### FOG detection latency

With the 2-layer LSTM model (16 units) in one-freezer-held-out cross validation, 95% (343 of 361) of freeze episodes were detected correctly (Table 7). The FOG episodes that were not detected were from Participant 7 (17 undetected episodes, of 221 total) and Participants 6 and 8 (one undetected episode). The model achieved a maximum 0.1 s (SD 0.32 s) average freeze detection latency, which occurred for Participant 7. A negative freeze detection latency means that, on average, freeze episodes were detected before the freeze started. Therefore, FOG episodes were detected on average before onset for Participants 1, 6, 8 and 9. FOG episodes were detected on average after the freeze had begun for Participants 2, 3 and 7.

**Table 7:** FOG detection latency (average and standard deviation) in one-freezer-held-out cross validation with the 2-layer LSTM model. A negative freeze detection latency means that the freeze was detected before the true freeze onset

Participant held out	Freezes correctly detected	Freezes not detected	Average FOG detection latency (s)
1	49	0	− 0.23 ± 0.55
2	35	0	0.02 ± 0.17
3	14	0	0.08 ± 0.25
6	9	1	− 0.04 ± 0.36
7	204	17	0.10 ± 0.32
8	23	1	− 0.55 ± 0.85
9	9	0	− 0.47 ± 0.74
Total	343	18	

### False positive classification and undetected freeze episodes

Of all false positives, 35.13% occurred during walking, 27.91% turning, and 34.74% standing. The remaining 2.22% of the false positives were during periods of undefined gait. *Undefined* refers to the beginning and end of a trial when no specific activity was being performed. For the false negatives, 58.67% of misclassifications occurred during turning and 46.12% occurred during walking.

Of all data labeled as standing, 65.3% were false positives. The percentages of false positives for walking (3.72%) and turning (7.09%) were much smaller. Almost no standing data were included in the final training set due to the data setup method (i.e., only freeze episodes and data before and after the freeze episodes were included in the training set). An activity recognition algorithm (not developed or implemented in this research) could be used as a first step of FOG detection to determine if the person is standing or walking prior to FOG classification. Since most of the standing data were misclassified as freeze (65.3% of standing data were false positive), an activity recognition algorithm could be applied to remove standing data, and the FOG detection algorithm would then only have to detect freezes during walking and turning, thereby reducing the number of false positives. To simulate this method, the 2-layer LSTM model was also evaluated on only active states (turning and walking) and excluding standing. In one-freezer-held-out cross validation for the 2-layer LSTM model, the mean specificity increased by 3.8%, from 89.5% (SD 3.6%) to 93.3% (SD 4.0%), when classifying active states only, compared to classifying both active and inactive states, while the sensitivity decreased negligibly by 0.5% (Table 8). For all-non-freezer-held-out validation, specificity increased from 81.6% to 88.4% (6.8% increase).

**Table 8:** FOG Detection: One-freezer-held-out cross validation using the 2-layer LSTM model on only active states and on both active and inactive states

Participant held out	Only active states		Both active and inactive states	
	Sensitivity (%)	Specificity (%)	Sensitivity (%)	Specificity (%)
1	79.2	95.8	83.0	92.5
2	77.2	96.8	77.2	90.3
3	72.5	93.6	72.5	92.8
6	85.9	94.8	85.9	90.2
7	77.9	92.3	77.4	89.5
8	86.2	84.2	86.2	81.2
9	92.2	95.4	92.2	89.8
Mean ± SD	81.6 ± 6.3	93.3 ± 4.0	82.1 ± 6.2	89.5 ± 3.6

Active states include walking and turning but exclude standing

### Percentage of time frozen

FOG detection models have been evaluated using the percentage of time frozen during a walking trial to compare the model classifications to the ground truth labels [46], and to compare labels from different labelers [47].

The percentage of time frozen was calculated for each session in the test set using all datapoints, whether FOG was correctly or incorrectly classified. The confusion matrix for each fold of the one-freezer-held-out cross validation was used to calculate the percentage of time frozen as follows:

$$Model\,percent\,time\,frozen= \frac{TP+FP}{\left(TP+TN+FP+FN\right)}$$

$$True\,percent\,time\,frozen= \frac{TP+FN}{(TP+TN+FP+FN)}$$

where TP is true positive, TN is true negative, FP is false positive, and FN is false negative. Percentage of time frozen results for the 2-layer FOG detection model are presented in Table 9. For the all-non-freezer-held-out testing, the model percent time frozen was 18.4%.

**Table 9:** Percentage of time frozen in one-freezer-held-out cross validation with the 2-layer LSTM model for FOG detection

Participant held out	True positive	True negative	False positive	False negative	Model time frozen (%)	True time frozen (%)
1	2868	76,729	6214	586	10.5	4.0
2	7154	99,394	10,736	2116	15.0	7.8
3	1089	131,869	10,288	413	7.9	1.0
6	3642	172,641	18,668	598	11.4	2.2
7	26,201	143,062	16,725	7640	22.2	17.5
8	3137	82,302	19,113	503	21.2	3.5
9	630	126,898	14,475	53	10.6	0.5
Mean ± SD					14.1 ± 5.2	5.2 ± 5.5

The model overestimated the percent time frozen. Since, percentage of time frozen is a generalized measure across an entire walking trial and does not indicate whether the classifications and true labels coincide, this measure does not relate to model viability in a cueing system. In a cueing system application, only the first datapoint(s) of a freeze needs to be correctly classified to appropriately deliver the cue. This research therefore used detection latency, together with common evaluation metrics such as sensitivity and specificity.

### FOG prediction

The best mean sensitivity and specificity were obtained after 4 training epochs for the 2-layer LSTM model and after 3 training epochs for the 3-layer LSTM model. The 2-layer LTSM model achieved 79.9% mean sensitivity (SD 10.3%) and 76.3% mean specificity (SD 9.0%) in one-freezer-held-out cross validation (Table 10). The same model achieved a higher mean specificity of 84.6% for all-non-freezer-held-out validation. For the 3-layer LSTM model, mean sensitivity 72.5% (SD 13.6%) was lower than for the 2-layer model, and mean specificity 81.2% (SD 6.8%) was higher (Table 11). However, the 3-layer model mean specificity for participants who did not freeze decreased to 69.5%. The FOG prediction model performance suggests that there may be patterns in the plantar pressure data indicative of upcoming FOG. The 2-layer model achieved 21.7% (SD 16.5%) precision and 0.31 (SD 0.19) F1 score. The 3-layer model achieved 25.5% (SD 19.4%) precision and 0.33 (SD 0.20) F1 score. Similar to FOG detection, the low values of precision and F1 can be attributed, in part, to the data imbalance in the test set. For example, for Participant P01, the 2-layer detection model correctly classified 2,868 of 3,454 FOG datapoints and correctly classified 76,729 of 82,943 Non-FOG datapoints, resulting in 83% sensitivity and 92.5% specificity. The model produced 2868 true positives and 6214 false positives. Thus, even though the model correctly identified 83% of the FOG datapoints and the false positives accounted for only 7.5% of the total Non-FOG datapoints, the precision was poor (31.6%) because of the high number of Non-FOG datapoints compared to FOG datapoints. In contrast, the 2-layer detection model produced a much higher precision for Participant P07 (61%) despite lower sensitivity (77.45) and specificity (89.5%). For Participant P07, 77.4% of the FOG datapoints were correctly classified, and 10.5% of the Non-FOG datapoints were misclassified.

**Table 10:** FOG Prediction: One-freezer-held-out cross validation with the 2-layer LSTM model after 4 training epochs

Participant held out	Sensitivity (%)	Specificity (%)	Precision (%)	F1 score
1	82.5	60.2	15.3	0.26
2	62.7	83.9	36.0	0.46
3	68.1	71.8	4.8	0.09
6	78.8	88.6	18.4	0.30
7	84.1	71.6	54.6	0.66
8	90.8	74.4	17.7	0.30
9	92.6	83.4	5.2	0.10
Mean ± SD	79.9 ± 10.3	76.3 ± 9.0	21.7 ± 16.5	0.31 ± 0.19

**Table 11:** FOG Prediction: One-freezer-held-out cross validation with the 3-layer LSTM model after 3 training epochs

Participant held out	Sensitivity (%)	Specificity (%)	Precision (%)	F1 score
1	67.4	90.3	37.6	0.48
2	52.8	88.6	40.2	0.46
3	57.6	75.2	4.6	0.09
6	80.4	86.3	16.0	0.27
7	72.9	80.2	59.9	0.66
8	81.0	76.7	17.4	0.29
9	95.4	71.2	3.1	0.06
Mean ± SD	72.5 ± 13.6	81.2 ± 6.8	25.5 ± 19.4	0.33 ± 0.20

### Most frequent freezer

One participant (P07) accounted for the majority of the FOG episodes in the dataset. To explore the generalizability of the model without Participant P07, the FOG detection model training and testing were re-run without Participant P07. The results are presented in Tables 12 and 13.

**Table 12:** FOG detection: One-freezer-held-out cross validation for the 2-layer LSTM model with and without most frequent freezer

	With P07’s data in training set		Without P07’s data in training set	
Held out participant	Sensitivity (%)	Specificity (%)	Sensitivity (%)	Specificity (%)
1	83.0	92.5	42.2	93.8
2	77.2	90.3	82.3	90.0
3	72.5	92.8	61.1	95.0
6	85.9	90.2	63.5	94.4
8	86.2	81.2	81.4	85.8
9	92.2	89.8	92.4	87.4
Mean ± SD	82.8 ± 6.4	89.5 ± 3.9	70.5 ± 16.7	91.1 ± 3.6

**Table 13:** FOG detection: One-freezer-held-out cross validation for the 3-layer LSTM model with and without most frequent freezer

	With P07’s data in training set		Without P07’s data in training set	
Held out participant	Sensitivity (%)	Specificity (%)	Sensitivity (%)	Specificity (%)
1	83.6	86.3	71.3	93.1
2	83.5	90.1	63.8	91.4
3	71.7	92.0	60.1	94.3
6	86.9	90.7	78.1	92.8
8	87.6	74.7	88.2	79.5
9	93.6	88.6	99.7	86.8
Mean ± SD	84.5 ± 6.6	87.1 ± 5.8	76.9 ± 13.7	89.7 ± 5.1

For FOG detection, the mean sensitivity for participants who froze (except P07) decreased when P07’s data were excluded (Tables 12 and 13). The decrease in sensitivity for most participants can be attributed to the decrease in available training instances in the absence of P07’s data. The model performed well when P07’s data were removed and included, which suggests that the model is likely not overtrained from P07’s data. Since more data for training typically improves model generalizability and helps prevent overfitting, data from all participants were included in this research.

## Discussion

### FOG detection

The new method for FOG detection using plantar pressure data detected 95% of freeze episodes. The proposed LSTM model used participant-independent LOPO cross validation, which ensured good generalizability compared to the LSTM network for FOG detection used in [17], which was not validated on data from an unseen participant (i.e., a participant whose data was not used for training). More data from people with PD who experience FOG may further improve the generalizability of the proposed method.

The 2-layer LSTM FOG detection model can be viably used in a real-time system since only 16 plantar-pressure features were used after z-score normalization, without signal filtering. The 2-layer LSTM model needs 51 KB computer memory and can be stored on a microcontroller. Compared to SVM models [19, 34] and a 1D CNN model [21], the new 2-layer LSTM model needs less computer memory (Table 14). The 2-layer LSTM model achieved shorter freeze detection latency than a C4.5 decision tree with 1 s windows [16]. The decision tree had 0.235 s (SD 0.175 s) mean freeze detection latency compared to the new 2-layer LSTM model’s 0.1 s (SD 0.3 s) maximum freeze detection latency.

**Table 14:** Computer memory requirement of different models

Model	Computer memory requirement (KB)
SVM [34]	1600
SVM [19]	1490
1D CNN [21]	145
New 2-layer LSTM	51

Compared to the SVM model in [19], which achieved 84.49% sensitivity and 85.83% specificity on 15 participants in LOPO cross validation, the new 2-layer LSTM model in this research produced slightly better specificity (89.5% (SD 3.6%)) but slightly worse sensitivity (82.1% (SD 6.2%)). The SVM model in [19] used 28 features from 1.6 s windows of IMU data after low pass filtering and required 1.6 MB of computer memory compared to the LSTM model’s 16 plantar-pressure features calculated without signal filtering. SVM model standard deviations were not reported in [19]; however, the sample size of 15 PD participants who froze was larger than the sample size of the new LSTM model, which included 7 PD participants who froze.

When classifying only active states, the new 2-layer LSTM model achieved better sensitivity (81.6% (SD 6.3%)) and specificity (93.3% (SD 4.0%)) than the 4-layer 1D CNN model in [43], which achieved 74.43% (SD 9.79%) sensitivity and 90.59% (SD 6.4%) specificity on only active states, (i.e., after removing standing segments) in LOPO cross validation on 8 participants. The 4-layer 1D CNN was trained with a batch size of 128 and may have poorer generalization compared to the new 2-layer LSTM model, which uses a batch size of 1. The 4-layer 1D CNN in [43] required accelerometers on the participant’s ankle, knee, and hip, which may be more obtrusive compared to the use of in-shoe plantar-pressure insoles. While the plantar-pressure insoles used in this research have wires and cuffs that could be considered obtrusive, wireless plantar-pressure measurement technology is available [48] that would eliminate this issue.

A 4-layer 1D CNN model that used 9-channel IMU data from 21 participants achieved better sensitivity (91.9%) and similar specificity (89.5%) on 4 held out participants [21] than the new 2-layer LSTM model. However, the 1D CNN model required 145 KB computer memory. The 1D CNN model used a batch size of 16, which may lead to slightly poorer generalization compared to the 2-layer LSTM model. The 1D CNN model combined information from 2 consecutive 2.56 s windows in the frequency domain before classification, which increased computational cost.

A 2-layer 1D CNN FOG detection model achieved slightly better sensitivity (83%) and lower specificity (88%) in a LOPO cross validation using acceleration data from a wrist mounted IMU [49], than the new 2-layer LSTM model in this paper. The CNN model used windows-based classification of data from 11 participants (184 FOG episodes) who froze, compared to our data from 7 participants (361 FOG episodes) who froze. The CNN model had a higher 0.25 s freeze detection latency (since 0.25 s windows were used) compared to the 2-layer LSTM model’s maximum 0.1 s (SD 0.32 s) average freeze detection latency.

The k-means clustering algorithm in [50] achieved a better sensitivity (92.4%) and specificity (94.9%) in a LOPO cross validation setting than the new LSTM model in this research. Entropy was extracted from 1 s sliding windows (with 0.5 s overlap) of raw acceleration data from 10 PD participants. However, in a k-means clustering algorithm, outliers should be removed in advance. Further research on a larger dataset is required to validate the utility of unsupervised learning algorithms (i.e., learning algorithms which use unlabelled data), such as k-means clustering, for FOG detection.

### FOG prediction

The new 3-layer LSTM model for FOG prediction using features extracted from plantar pressure data achieved good specificity; however, improvement in sensitivity is desired. The new 3-layer LSTM model, with 72.5% mean sensitivity (SD 13.6%) and 81.2% mean specificity (SD 6.8%) performed similarly to a multilayer perceptron neural network trained to predict FOG using electroencephalogram (EEG) data [39]. The multilayer perceptron neural network achieved 73.19% mean sensitivity and 80.16% mean specificity in classifying data between 5 and 1 s before freeze onset for five held out participants using wavelet energy [39]. A backpropagation neural network using EEG signals from 16 participants predicted FOG with 85.56% sensitivity and 80.25% specificity on five held-out participants [42]. This result had better sensitivity and similar specificity compared to the new 3-layer LSTM prediction model. Since EEG signal artifacts were removed with visual inspection and the signal needed to be filtered [42], the noise and complex pre-processing make the EEG approach challenging for use in a wearable system, in which computational power is limited.

Most FOG prediction models in the literature have used all participant’s data in both training and test sets. For example, in [40] a 2-layer LSTM network was trained using half of the data from each participant and was tested on the other half. While the model achieved 87.54% accuracy in FOG prediction with 1 s Pre-FOG duration and 85.54% accuracy with 2 s Pre-FOG duration [40], the results cannot be generalized on new participants because all participant’s data were used in both training and validation sets. Furthermore, the 2-layer LSTM network in [40] will likely lead to poor generalization, since it was trained using a large batch size of 1000 compared to the new 3-layer LSTM model’s batch size of 1.

A FOG prediction model achieved a higher 84.1% sensitivity and 85.9% specificity for FOG prediction in LOPO cross validation with 2 s Pre-FOG length [51] than the new 3-layer LSTM model. The model used angular velocity signals from 11 PD participants equipped with IMU sensors placed on the shins. However, with use of complex preprocessing steps, such as step segmentation and features extracted in both the time and frequency domains, this approach would be more challenging to implement in real time than the new 3-layer LSTM model.

The FOG detection and prediction models developed in this research used very little computer memory and therefore may be suitable for real-life systems that use wearable microprocessors with limited computational resources. Since the plantar-pressure insole sensors used in this research are meant to be single use, a different insole sensor would be required for a real-life wearable system. The current models were developed using data from seven participants who froze; further study with larger datasets is planned.

## Conclusion

For FOG detection, a 2-layer LSTM model achieved results comparable to existing literature. This research showed that an LSTM model with features extracted from plantar pressure data can be used for FOG detection. The research also showed that FOG detection models could move away from the window-based approach, which could save critical time in real-time implementation without compromising freeze detection performance.

For FOG detection, most inactive-state data (standing) were misclassified by the model. When classifying only active states, the new LSTM model specificity increased by 3.8% while the sensitivity was hardly affected. Coupling FOG detection with an activity recognition system could decrease false positives, while correctly detected FOG would remain unaffected.

For FOG prediction, the new 3-layer LSTM model achieved greater specificity (81.2%, SD 6.8%) but lower sensitivity (72.5%, SD 13.6%) than most person-independent models in the literature. However, the new LSTM model in this research was more generalizable since the results were obtained in a participant-held-out cross-validation setting, compared to the literature, where models were frequently validated on held out participants without cross validation. The new LSTM models used plantar pressure data without any signal filtering, which reduces the complexity of the model and could save valuable time in a real-time system.

## Future work

To improve the precision, the number of false positives produced by the models should be minimized. There are several ways in which this can be achieved. For instance, a state recognition system could be implemented to automatically detect when a person is walking or not. In addition, if used as part of a cueing system, a consecutive cue threshold could be implemented. The models presented in this paper classified every datapoint separately; however, in a real-time cuing system, a minimum number of classifications could be required to activate a cue. Given the resolution of 100 Hz used in this paper, requiring several consecutive classifications to trigger a cue would likely result in an unnoticeable delay.

The training dataset did not contain standing activity data, thus the standing data (inactive state) in the validation set were misclassified as FOG by the model. While classifying only active states improved model performance, the model was not trained to recognize FOG during gait initiation. If used in a real-life system, the model may be unable to detect all FOG episodes. Given the different characteristics of FOG that occur during gait initiation and during walking, separate models may perform better than a single model for all FOG. Further study could include one FOG detection model for gait initiation and one for walking.

To improve LSTM model performance, more features could be explored together with a feature-selection technique, which would select the best features to be used in models. Models based on time series prediction could also be explored to improve FOG prediction performance.

A personalized model could be made with transfer learning. The final few layer’s weights in a participant-independent deep-learning model could be trained with the target participant’s data. In addition to training the model with a larger dataset, future work could implement the LSTM model in a microcontroller for real-time FOG detection and prediction.

## Data Availability

The datasets used and analysed during the current study are available from the corresponding author on reasonable request.

## Abbreviations

FOG: Freezing of gait
PD: Parkinson’s disease
UPDRS: Unified Parkinson’s disease rating scale
Pre-FOG: Time or data immediately preceding a FOG episode
Non-FOG: Time or data that does not contain FOG
LSTM: Long short-term memory (type of neural network)
CNN: Convolutional neural network
SVM: Support vector machine classifier
EEG: Electroencephalogram
IMU: Inertial measurement unit
RNN: Recurrent neural network
LOPO: Leave-one-participant out (type of cross validation)
COP: Centre of pressure
GRF: Ground reaction force
SD: Standard deviation
